# Loss of adenosine A3 receptors accelerates skeletal muscle regeneration in mice following cardiotoxin-induced injury

**DOI:** 10.1038/s41419-023-06228-7

**Published:** 2023-10-28

**Authors:** Nastaran Tarban, Albert Bálint Papp, Dávid Deák, Péter Szentesi, Hajnalka Halász, Andreas Patsalos, László Csernoch, Zsolt Sarang, Zsuzsa Szondy

**Affiliations:** 1 Doctoral School of Molecular Cell and Immune Biology, University of Debrecen, Debrecen, Hungary; 2 Doctoral School of Dental Sciences, University of Debrecen, Debrecen, Hungary; 3 Laboratory Animal Facility, Life Science Building, University of Debrecen, Debrecen, Hungary; 4 Department of Physiology, Faculty of Medicine, University of Debrecen, Debrecen, Hungary; 5 Departments of Medicine and Biological Chemistry, Johns Hopkins University School of Medicine, Institute for Fundamental Biomedical Research, Johns Hopkins All Children’s Hospital, St, Petersburg, FL USA; 6 Department of Biochemistry and Molecular Biology, Faculty of Medicine, University of Debrecen, Debrecen, Hungary; 7 Division of Dental Biochemistry, Department of Basic Medical Sciences, Faculty of Dentistry, University of Debrecen, Debrecen, Hungary

**Keywords:** Adaptive immunity, Cell signalling, Trauma

## Abstract

Skeletal muscle regeneration is a complex process orchestrated by multiple interacting steps. An increasing number of reports indicate that inflammatory responses play a central role in linking initial muscle injury responses to timely muscle regeneration following injury. The nucleoside adenosine has been known for a long time as an endogenously produced anti-inflammatory molecule that is generated in high amounts during tissue injury. It mediates its physiological effects via four types of adenosine receptors. From these, adenosine A3 receptors (A3Rs) are not expressed by the skeletal muscle but are present on the surface of various inflammatory cells. In the present paper, the effect of the loss of A3Rs was investigated on the regeneration of the tibialis anterior (TA) muscle in mice following cardiotoxin-induced injury. Here we report that regeneration of the skeletal muscle from A3R^−/−^ mice is characterized by a stronger initial inflammatory response resulting in a larger number of transmigrating inflammatory cells to the injury site, faster clearance of cell debris, enhanced proliferation and faster differentiation of the satellite cells (the muscle stem cells), and increased fusion of the generated myoblasts. This leads to accelerated skeletal muscle tissue repair and the formation of larger myofibers. Though the infiltrating immune cells expressed A3Rs and showed an increased inflammatory profile in the injured A3R^−/−^ muscles, bone marrow transplantation experiments revealed that the increased response of the tissue-resident cells to tissue injury is responsible for the observed phenomenon. Altogether our data indicate that A3Rs are negative regulators of injury-related regenerative inflammation and consequently also that of the muscle fiber growth in the TA muscle. Thus, inhibiting A3Rs might have a therapeutic value during skeletal muscle regeneration following injury.

## Introduction

Skeletal muscle is one of the few organs which is capable of full regeneration following injury [1]. Though the skeletal muscle stem cell, the satellite cell (SC), is essential for the regeneration process, its activation and differentiation require its continuous interaction with various cell types present or appearing at the site of injury [2]. The regeneration process begins with the degeneration of myofibers which release various danger-associated molecules (DAMPs) recognized by cells within the skeletal muscle tissue, such as tissue-resident macrophages (MΦs) [3] and mast cells [4]. In response, mast cells degranulate [4], while tissue-resident MΦs actively produce chemoattractants such as macrophage-inflammatory protein (MIP)-1, MIP-2 and keratinocyte chemoattractant (KC) to recruit neutrophils [5] and further mast cells [6] creating an inflammatory environment [7, 8]. These infiltrating cells start to clear the damaged myofibers at the injury site. Meanwhile, they also secrete various types of cytokines to recruit more immune cells such as Ly6C^high^ monocytes which, once appear, start their differentiation into inflammatory Ly6C^high^ MΦs [2]. Infiltrating inflammatory Ly6C^high^ MΦs are the main players of cell clearing and release further pro-inflammatory cytokines contributing to the inflammatory environment needed for promoting SC proliferation [7, 8]. Inflammation plays such a determining role in skeletal muscle regeneration that inhibition of it completely destroys muscle repair [3, 4].

Tissue-resident MΦs and mast cells not only recruit inflammatory cells but also play a central role in initiating the activation of SCs by releasing nicotinamide phosphoribosyltransferase (NAMPT) [9], and tumor necrosis factor (TNF)-α, tryptase or histamine [10], respectively. These molecules trigger the asymmetric cell division and differentiation of SCs into myoblasts which also proliferate and fuse together to form the new myofibers. While the pro-inflammatory cytokines TNF-α, interleukin-1β (IL-1β), and IL-6 promote SC proliferation [11], the differentiation or fusion of myoblasts are driven by growth factors like transforming growth factor (TGF)-β [12], insulin-like growth factor (IGF)-1 [13] or growth differentiation factor 3 (GDF3) [14] produced by reparative Ly6C^low^ MΦs that are generated from the Ly6C^high^ MΦs during the efferocytosis process [15]. This M1/M2–(pro-inflammatory/anti-inflammatory) like phenotypic conversion is further accelerated by IL-10 and IL-4 produced by the already generated Ly6C^low^ MΦs facilitating this way skeletal muscle repair [16, 17]. Several cytokines (such as TNF-α, TGF-β, GDF3, and IGF-1) are also produced by the myoblasts regulating their proliferation and differentiation in an autocrine way. From these, TNF-α and GDF3 were shown to inhibit the early differentiation of myoblasts, and their silencing resulted in an increase in every myotube parameter tested [18].

The composition of the appearing reparative Ly6C^low^ MΦs is dynamically changing during the repair process, and they can be divided at least into three subgroups: growth factor-expressing, resolution-related and antigen-presenting cells [19]. Since some of them specifically express certain genes, the loss of the proteins encoded by them individually affects the further conversion (for example the conversion into CD206^+^ cells) of the different reparative macrophage subpopulations [20–22].

The nucleoside adenosine has been known for a long time as an endogenously produced anti-inflammatory molecule [23]. It is produced by cells from the breakdown of adenosine nucleoside triphosphate (ATP) both intracellularly and extracellularly, in response to stress. Following injury, a sharp increase in adenosine production is expected (from the resting nanomolar concentrations to micromolar concentrations [23]). Adenosine is then either taken up by the surrounding cells or degraded to inosine by the adenosine deaminase enzyme [24]. Adenosine has been shown to mediate its effects via four types of adenosine receptors, which are all 7-transmembrane receptors but differ in their affinity for adenosine, in the G protein they interact with, and in the signaling pathways they activate. Since these receptors are expressed by nearly all inflammatory cells, adenosine is capable of affecting every aspect of inflammation. The anti-inflammatory effects of adenosine have been shown to be mediated primarily via the adenosine A2 receptors (A2Rs) [23]. These receptors are coupled to G_s_ and trigger increases in cAMP levels [23]. Adenosine A2A receptors (A2ARs) are stimulated by 5 × 10^−7^ M, while adenosine A2B receptors (A2BRs) by 1 × 10^−5^ M concentrations of adenosine, respectively. Activation of A2Rs was demonstrated to inhibit neutrophil transmigration and the production of pro-inflammatory cytokines by neutrophils or by inflammatory macrophages while promoting M1/M2 conversion of macrophages and the production of angiogenic factors by them [23].

Unlike the role of A2Rs in the regulation of inflammation, the involvement of adenosine A3 receptors (A3Rs) is more enigmatic [25]. A3Rs are also G protein-coupled receptors stimulated by 10^−6^ M concentrations of adenosine, but unlike A2Rs, they activate G_i_ and consequently decrease the adenylate cyclase activity [26]. Thus, one would expect that they interfere with the anti-inflammatory effects of A2Rs. Still, the observation is that the effect of A3Rs on inflammation is cell type-specific and very often anti-inflammatory. For example, A3R stimulation inhibits the IL-1β, TNF-α, MIP-1α, interferon regulatory factor 1, and inducible nitric oxide synthase gene expressions in human and murine macrophage cell lines [25]. This is attributed to the fact that in addition to inhibiting the adenylate cyclase pathway, A3Rs acting via G_q_ are capable of triggering the phospholipase C and Akt signaling pathways as well [26]. In addition, they selectively can be activated by inosine, the breakdown product of adenosine [27] indicating a more prolonged activation of this receptor during inflammation.

Since inflammation plays a central role in orchestrating skeletal muscle repair [3, 4], we decided to investigate the effect of the loss of A3Rs on the inflammatory response and on the skeletal muscle regeneration by studying the tibialis anterior muscle of mice following cardiotoxin-induced injury.

## Materials and methods

### Materials

All reagents were obtained from Merck (Darmstadt, Germany) except when indicated otherwise.

### Experimental animals

Experiments were carried out using 3- to 6-month-old young adult male C57BL/6J A3R^+/+^ mice and their full-body A3R^−/−^ littermates [28]. Mice were bred in the heterozygous form under specific pathogen-free conditions in the central animal facility of the University of Debrecen.

### In vivo assessment of muscle force

The force of the fore paw was measured as described previously [29]. In brief, once the animals consistently grasped the grip test meter bar, they were gently pushed away from the instrument in a horizontal direction. The maximum force that the animal applied before releasing the bar was recorded and digitized at a 2 kHz rate. The test was repeated 10–15 times in order to collect one data point for each mouse. For all animal groups, the grip test was conducted on the day the animals were sacrificed.

### Voluntary running

Mice were singly housed in a cage with a mouse running wheel (Campden Instruments Ltd., Loughborough, UK). Wheels were connected to a computer, and revolutions were recorded in 20-min intervals, nonstop for 14 days. The average and the maximal speed, the covered distance and the duration of running were calculated daily for each individual mouse and then averaged by groups.

### Forced treadmill running

The time and distance to exhaustion of mice were evaluated during treadmill running on a motor-driven wheel-track treadmill. The speed of running started at 1 km/h and increased by 0.1 km/h every 2 min at 0° tilt angle until the exhaustion of mice was reached.

### Ex vivo assessment of muscle force

Muscle contraction was measured as previously described [29]. Briefly, the examined muscles were removed manually and placed horizontally in an experimental chamber continuously superfused (10 ml/min) with Krebs solution (containing in mM: NaCl 135, KCl 5, CaCl_2_ 2.5, MgSO_4_ 1, Hepes 10, NaHCO_3_ 10, and glucose 10; pH 7.2; room temperature) equilibrated with 95% O_2_ plus 5% CO_2_. On one end, the muscle was connected to a rod, and on the other, to a capacitive mechano-electric force transducer. Two platinum electrodes were inserted beneath the muscle to deliver short, supramaximal pulses of 2 ms duration to induce single twitches. The digitalization of force responses at 2 kHz was carried out using Digidata 1200A/D interface, and the acquired data were analyzed with AxoTape software (Axon Instruments, Foster City, CA, USA). Muscles were then stretched by moving the transducer to a length that provided the greatest force response and allowed to equilibrate for 5 min. Single pulses at 0.5 Hz were used to induce single twitches. At least ten twitches were recorded from each muscle under these conditions. Individual force transients within such a train varied in amplitude by less than 3%; thus, the mean amplitude of all transients was used to characterize the muscles. Tetanus was triggered using single pulses at 200 Hz for 200 ms (EDL) or 100 Hz for 500 ms (SOL). The duration of individual twitches and tetani was calculated using the time between the onset of the transient and the relaxation to 10% of the maximal force.

### Cardiotoxin-induced muscle injury model

Mice were anesthetized with 2.5% isoflurane using a SomnoSuite device. To induce muscle damage, 50 μl of 12 μM cardiotoxin (CTX) (Latoxan, Valence, France), dissolved in phosphate-buffered saline (PBS) was injected into the tibialis anterior (TA) muscle. The size of the control and treated groups was the same as reported by others in similar experiments [30]. There were no inclusion or exclusion criteria used in the selection of the animals. Animals from each cage were randomly allocated to the control or treated groups, but no blinding was used. Muscles were harvested at various time points following injury, weighed, and processed for further experiments. In some experiments, mice were co-injected either with 2 mg/kg MRS1191, an A3R antagonist (MedChemExpress, Monmouth Junction, NJ, USA), or 100 μg/kg IB- MECA, an A3R agonist (MedChemExpress).

### Bone marrow transplantation

Recipient Pep/BoyJ, A3R null, A3R^+/+^ wild-type mice (7-week-old males) were irradiated with 11 Gy using a Theratron 780C cobalt unit for the ablation of the recipient bone marrow. The animals to be irradiated were immobilized using a circular cage (mouse pie cage) that could hold up to 11 mice. Following irradiation, isolated bone marrow cells (in sterile RPMI-1640 medium) flushed out the femur, tibia, and humerus from donor Pep/BoyJ, A3R null, A3R^+/+^ mice were transplanted into the recipient mice by retro-orbital injection (20 × 10^6^ bone marrow (BM) cells per mouse). After 30 days of recovery, TA muscle injury was induced by CTX injection. The utilization of the CD45 congenic model allowed us to detect donor, competitor, and host contributions in hematopoiesis and repopulation efficiency of donor cells (congenic mice with CD45.1 versus CD45.2). The CD45.1 and CD45.2 cell contributions were detected by flow cytometry 22 days after muscle injury induction. Mice were sacrificed by isoflurane overdose, muscle TA muscles were collected for histological analysis, and blood was drained from the heart into heparinized tubes. Red blood cells were lysed by mixing 100 μl blood with 1 ml of ACK hypotonic lysis buffer (Thermo Fisher Scientific, Waltham, MA, USA) for 5 min. After the removal of red blood cells, the remaining cells were suspended in 50 μl PBS-50% FBS and stained by 2 μl anti-mouse CD45.1-FITC (110705, clone A-20, BioLegend, San Diego, CA, USA) and 2 μl anti-mouse CD45.1-PE (111103, clone QA18A15, BioLegend) antibodies and incubated on ice for 30 min. Fluorescent intensity was measured on a Becton Dickinson FACSCalibur instrument (Becton, Dickinson and Company, Franklin Lakes, NJ, USA).

### Hematoxylin/eosin and immunofluorescent stainings of muscles

TA, extensor digitorum longus (EDL) and soleus (SOL) muscles from control mice or TA muscles at the indicated days post-injury were dissected for histological assessment. The muscles were snap-frozen in liquid nitrogen-cooled isopentane and kept at −80 °C. Seven-micrometer thick cryosections were cut at −20 °C using a 2800 Frigocut microtome (Leica, St Jouarre, France) and were kept at −20 °C until further analysis. Hematoxylin/eosin (H&E) staining was performed to assess the overall morphology and presence of necrotic fibers following injury. Images from the sections were taken using an AMG EVOS cl microscope (Thermo Fisher Scientific). To calculate the cross-sectional areas (CSA) and collagen-stained areas, frozen muscle sections were incubated in 10 mM citric acid-sodium citrate buffer (pH 6.0) for 15 min, then in blocking solution (50% FBS in PBS) for 1 h at room temperature. After blocking, samples were labeled with Dylight 488 conjugated anti-laminin B (PA5-22901, Invitrogen, Carlsbad, CA, USA) (1:100) and anti-collagen 1 antibody (SAB4500362) (1:100) at 4 °C overnight followed by Alexa Fluor 488 conjugated Goat anti-Rabbit IgG secondary antibody followed by washing three times with PBS. The nuclei were labeled with 4 μg/ml 4′,6-diamidino-2-phenylindole (DAPI) (Invitrogen), and the slides were mounted with glass coverslips. Images were taken on a FLoid Cell Imaging Station fluorescent microscope (Thermo Fisher Scientific) and analyzed using ImageJ v1.52 software (National Institutes of Health, Bethesda, MD, USA) with a muscle morphometry plugin. Areas containing fibers with centrally-located nuclei were considered regenerating muscle parts. CSAs are reported in μm^2^, while collagen content is reported as the percentage of the total examined regenerating area.

### Quantification of necrotic regions

Necrotic region was defined as an area containing myofibers infiltrated by basophil single cells and quantified as described previously [19]. Briefly, 4 non-overlapping microscope view field areas were digitally captured from 6–8 H&E stained TA muscle sections at 200-fold magnification. The percentage of necrotic area relative to the total regenerating area was calculated after manual outlining of the necrotic regions in the sections.

### Isolation of muscle-derived CD45^+^ and CD31^+^ cells

Muscle-derived cell isolation was carried out as described previously [20]. Briefly, control and regenerating TA muscles were removed and dissociated in RPMI containing 0.2% collagenase II (Thermo Fisher Scientific) at 37 °C for 1 h and filtered stepwise through 100 µm and 40 µm filters. CD45^+^ and CD31^+^ cells were labeled with microbeads and isolated using magnetic sorting columns (Miltenyi Biotec, Gladbach, Germany, catalog numbers: 130-052-301 and 130-097-418, respectively). The purity of isolated cells (around 95%) was determined by FACS analysis following staining them with phycoerythrin (PE)-conjugated antibodies against mouse CD45.2 and CD31 (109807 and 102407, BioLegend), respectively (Supplementary Fig. S1).

### Gene expression analysis

Total RNA of muscle-derived CD45^+^ cells and TA muscles homogenized in TRIzol with a Shakeman homogenizer (BioMedical Science, USA) was isolated with TRIzol reagent (Invitrogen), according to the manufacturer’s instructions. Total RNA was reverse transcribed into cDNA using a High Capacity cDNA Reverse Transcription Kit (Thermo Fisher Scientific), according to the manufacturer’s instructions. Quantitative RT-PCR was carried out in triplicate using FAM-labeled MGB assays (Thermo Fisher Scientific) on a Roche LightCycler LC 480 real-time PCR instrument (Roche, Basel, Switzerland). Relative mRNA levels in the case of CD45^+^ cells were calculated using the comparative C_T_ method and normalized to beta-actin (β-actin) mRNA. In the case of the TA muscle samples, gene expression was normalized to the total RNA content (200 ng) of the samples. Catalog numbers of the TaqMan assays used were the following: A3R Mm07296455_m1, A2AR Mm00802075_m1, A2BR Mm00839292_m1, CCL2/MCP-1 Mm00441242_m1, Tnf Mm00443258_m1, IL1B Mm00434228_m1, IL6 Mm00446190_m1, IGF1 Mm00439560_m1, Tgfb1 Mm01178820_m1, Gdf3 Mm00433563_m1, Pax7 Mm00834082_m1, MyoD1 Mm00440387_m1, Myog Mm00446194_m1, Nr4a1/Nur77 Mm01300401_m, and Actb Mm02619580_g1.

### Single-cell RNA sequencing and analysis of muscle-derived CD45^+^ cells

Single-cell gene expression barcode, feature, and count matrices from CD45^+^ cells isolated on day 4 following CTX-induced muscle injury were used from dataset GSE161467 [19]. Downstream analysis was carried out as described previously [22].

### Quantification of satellite cells in the TA muscle following CTX-induced injury

For intramuscular SC detection, TA was collected at 2, 3, and 4 days post-injury and dissociated in RPMI containing 0.2% collagenase II (Thermo Fisher Scientific) at 37 °C for 1 h and filtered through a 100 µm filter. Prior to staining, ~225,000pcs polystyrene microbeads (8 mm, 78511) were added to the muscle cell suspension samples to determine the absolute cell numbers later. The identification of the muscle precursor cells was based on the α7-integrin (PE-conjugated, 130-120-812, Miltenyi Biotec) staining of the SCs. Other muscle tissue-resident and immigrant cell types were gated from the measurement by specific staining with a moisture of monoclonal antibodies, including biotin anti-mouse CD45 (103104, BioLegend), biotin anti-mouse CD31 (102404, BioLegend), and biotin anti-mouse Ter119 (79748, BioLegend). In the second step, APC-conjugated streptavidin (405207, BioLegend) was added to the cells. Before the measurement, cells were washed with 0.5% BSA-physiological saline and suspended in 0.5% BSA-physiological saline supplemented with SYTO16 green-fluorescent nucleic acid stain (S7578, Invitrogen) (5000× dilution) and 7-AAD non-cell-permeable dead cell stain (A1310, Thermo Fisher) (1000× dilution) to exclude the injured and dead cells. The measurement was performed with a FACS Aria III cytometer (Becton, Dickinson and Company) equipped with violet (405 nm), blue (488 nm), yellow (561 nm), and red (633 nm) lasers. The measurement of the microbeads was based on their intense side directional light scattering (SSC) properties. The living cells were gated to CD45/CD31/Ter119 positive and negative populations according to their APC fluorescence. The APC non-stained cells mainly involved the integrin-α7^+^, Sca1^−^, and CD140a^−^ SC cells. The absolute cell count was based on the ratio of the cells of interest to the microbeads within the measured samples.

### Quantification of intramuscular immune cells by flow cytometry

The magnetically separated muscle-derived CD45^+^ cells were stained with Alexa Fluor 488-conjugated anti-F4/80 antibody (MF48020, Invitrogen) at room temperature for 15 min. The cells were gated based on their forward and side scatter characteristics. Macrophages were gated as F4/80^+^ cells. F4/80^+^ macrophages were also analyzed for expression of Ly6C, CD206, or major histocompatibility complex (MHC)II, following staining with the corresponding antibodies, Ly6C PerCP-Cy5.5 (128012, BioLegend), CD206-PE (141705, BioLegend), or MHCII-FITC (107605, BioLegend), respectively. Fluorescent intensity was measured on a Becton Dickinson FACSCalibur instrument (Becton, Dickinson and Company).

### Statistical analysis

All the data presented represent the results of at least three independent experiments, and all data are presented as mean and individual values or mean or median ±SD. Statistical analysis was performed using two-tailed, unpaired Student’s *t*-test and ANOVA with post-hoc Tukey HSD test. The equal variance of the sample groups was tested by an F-test. * denotes *p* < 0.05, ** denotes *p* < 0.01, *** denotes *p* < 0.001.

## Results

### A3R deficiency does not alter the efficiency of the skeletal muscle function

**Fig. 1:**
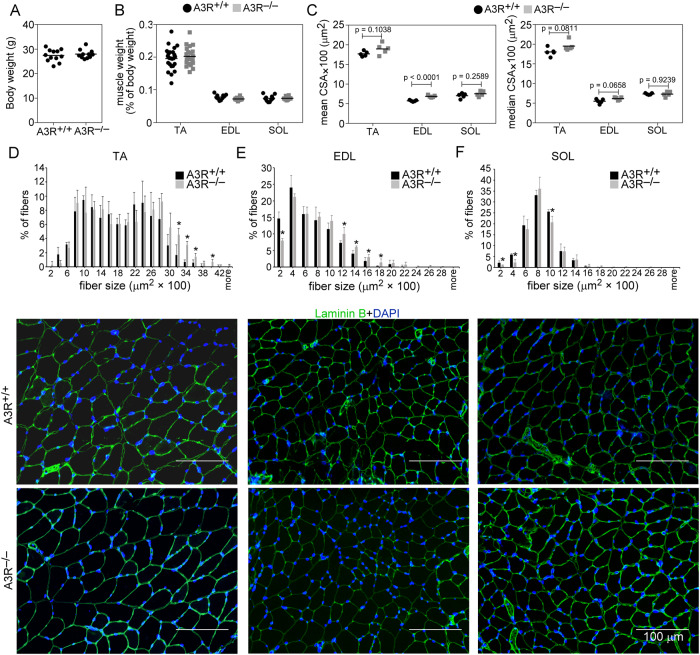
Phenotypic characteristics of TA, EDL, and SOL muscles in wild-type and A3R^−/−^ mice. **A** Body weight and **B** muscle weight/body weight ratios in A3R^+/+^ and A3R^−/−^ mice. **C** Mean and median myofiber cross‐sectional area (CSA) of the TA, EDL, and SOL muscles in A3R^+/+^ and A3R^−/−^ untreated mice. Data are expressed as mean and individual values, and statistical significance was determined by two-tailed Student’s *t-*test (*n* = at least 4 samples). Myofiber size repartition in **D** TA, **E** EDL, and **F** SOL control muscles of A3R^+/+^ and A3R^−/−^ mice with their representative immunofluorescence pictures of laminin (green) and DAPI (blue) nuclear staining. ImageJ software was used to examine 500 or more myofibers in each sample. Scale bars, 100 μm. Mean values ± SD are shown, and statistical significance was determined by two-tailed Student’s *t-*test. *p < 0.05. (*n* = 4 samples).

To study the role of A3Rs in muscle homeostasis, we compared the characteristics of the TA, EDL, and SOL muscles from A3R^+/+^ and A3R^−/−^ mice. There was no significant difference between the body weights, TA, EDL, and SOL muscle weights between A3R^+/+^ and A3R^−/−^ mice (Fig. 1A, B). However, a tendency for higher mean and median fiber CSA in the fast twitch muscles (TA, EDL) of A3R^−/−^ mice was detected as compared to that of the wild-type ones (Fig. 1C). In accordance, the percentage of bigger fibers was higher in the two fast twitch A3R^−/−^ muscles as compared to wild-type ones (Fig. 1D–F).

We also determined whether the loss of A3R has an impact on the physical performance of A3R^−/−^ mice. In vivo grip force is a measure of muscular strength and can be used as a tool to study the upper body and overall strength. Thus, we determined the grip force in 18- to 20-week-old A3R^+/+^ and A3R^−/−^ mice (Supplementary Table 1). We found that the maximal force of A3R^−/−^ animals was significantly smaller than that of A3R^+/+^ animals (161.94 ± 7.13 and 122.79 ± 3.44** mN in wild type versus A3R null muscles, respectively. Significantly different at *p* < 0.01, *n* = 5). Since the average body weight was identical in both groups, the normalized grip force was also significantly smaller in A3R^−/−^ animals (5.80 ± 0.27 and 4.57 ± 0.07** mN/g in wild type versus A3R null muscles, respectively. Significantly different at *p* < 0.01, *n* = 5). In accordance, we found a significant decrease also in the voluntary running of the A3R^−/−^ mice as compared to their wild-type controls (Supplementary Table 1). The daily running distance was not altered, but the average speed and the maximal speed were significantly lower. However, when the forced treadmill running of the animals was compared, no difference in their performance was found. Neither did we find a decrease in the maximal force capability of the isolated EDL or SOL muscles (Supplementary Table 2). These data indicate that the altered neurological functions [31], rather than an altered skeletal muscle function explain the worse grip force and voluntary running results of the A3R null animals.

### Accelerated TA muscle regeneration in A3R deficient mice

**Fig. 2:**
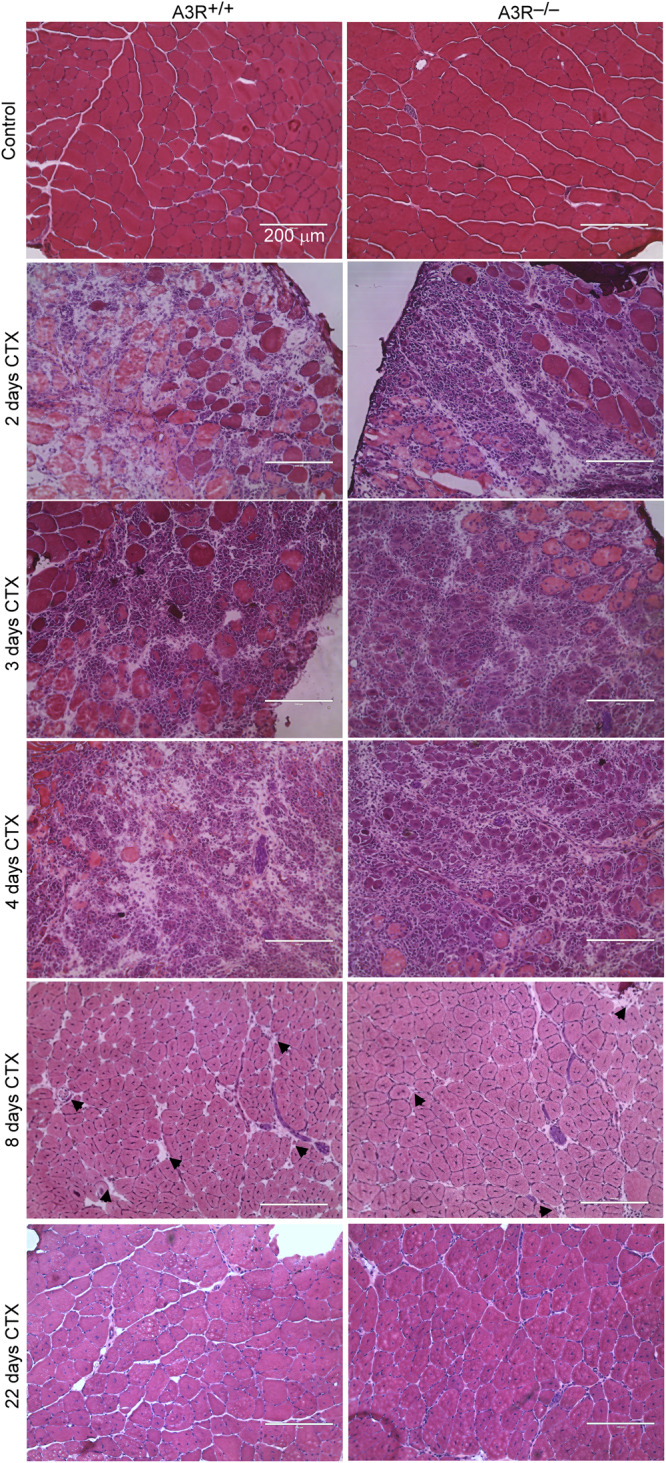
Time-dependent histological morphology of the TA muscles in A3R^+^^/+^ and A3R^−^^/^^−^ mice following CTX-induced injury. Injection of 50 μl of 12 μM CTX into the TA muscles was used to trigger muscle damage. Representative H&E‐stained cross‐sections of the TA muscles from A3R^+/+^ and A3R^−/−^ mice before (control) and at days 2, 3, 4, 8, and 22 days after CTX treatment (*n* = 4). Arrows indicate necrotic areas. Scale bars, 200 μm.

**Fig. 3:**
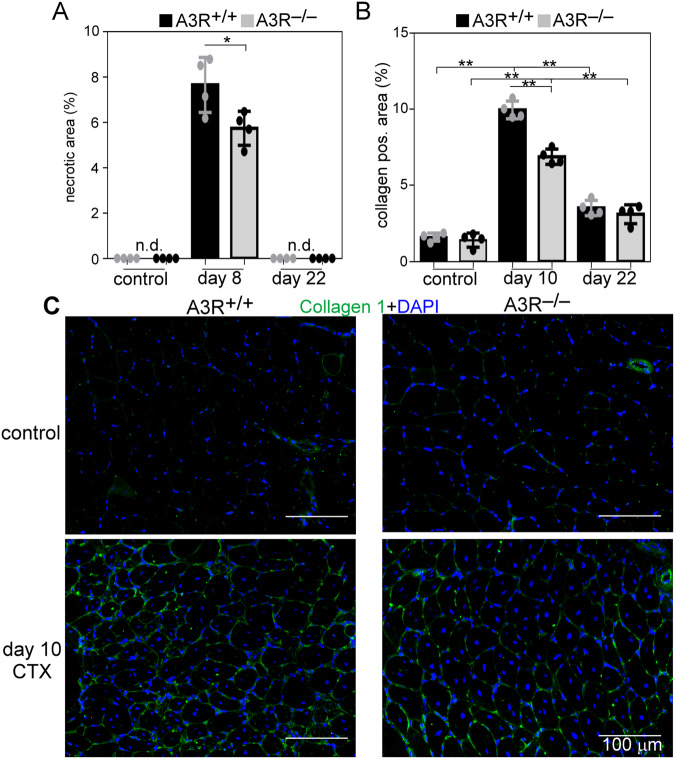
Enhanced dead cell clearance and decreased collagen deposition in the regenerating A3R^−/−^ TA muscles. **A** Necrotic regions in the control and regenerating muscles of A3R^+/+^ and A3R^−/−^ mice at days 0, 8, and 22 post‐CTX injury. **B** Quantification of collagen 1‐positive regions in control and 10‐ and 22‐day regenerating muscles of A3R^+/+^ and A3R^−/−^ mice. Data are expressed as mean and individual values and statistical significance was determined by two-tailed Student’s *t-*test and ANOVA. *p < 0.05, **p < 0.01. *n* = 4. n.d., non-detected. **C** Representative immunofluorescence pictures of type 1 collagen (green) and DAPI (blue) nuclear staining in control and 10‐day regenerating TA muscles of A3R^+/+^ and A3R^−/−^ mice. Scale bars, 100 μm.

To study the potential involvement of A3Rs in skeletal muscle regeneration, we performed histological analysis of control and CTX-injected TA muscles of wild-type and A3R^−/−^ mice. There was no obvious alteration in the gross appearance between the control muscles (Fig. 2). On days 2, 3, and 4 following injury both A3R^+/+^ and A3R^−/−^ regenerating muscles displayed local necrosis and abundant inflammatory cell infiltration. Previous studies indicated that impaired efferocytosis delays the presence of necrotic areas [20]. Interestingly, despite the fact that loss of A3Rs did not affect the efferocytosis by macrophages [32], more necrotic fibers were cleared from the A3R^−/−^ muscles by day 8 following CTX-induced injury (Figs. 2 and 3A). By day 22 post-injury, the overall histological architecture of both A3R^+/+^ and A3R^−/−^ muscles had been largely restored, and necrotic fibers were no longer visible (Figs. 2 and 3A).

In addition to inflammatory cells and SCs, efficient muscle regeneration also involves the appearance of fibroblasts which produce a new temporary extracellular matrix to stabilize the healing tissue and to provide a scaffold for the growing fibers. Collagen deposition first increases but then decreases with time [33]. Accordingly, by day ten following CTX-induced injury we detected an increased amount of collagen I in the regenerating muscles of both A3R^+/+^ and A3R^−/−^ mice as compared to their own non-regenerating muscles, but with significantly lower collagen deposition in the case of A3R^−/−^ muscles, while at 22-day post-injury, the collagen deposition returned close to normal and was very similar in the two types of muscles (Fig. 3B, C).

**Fig. 4:**
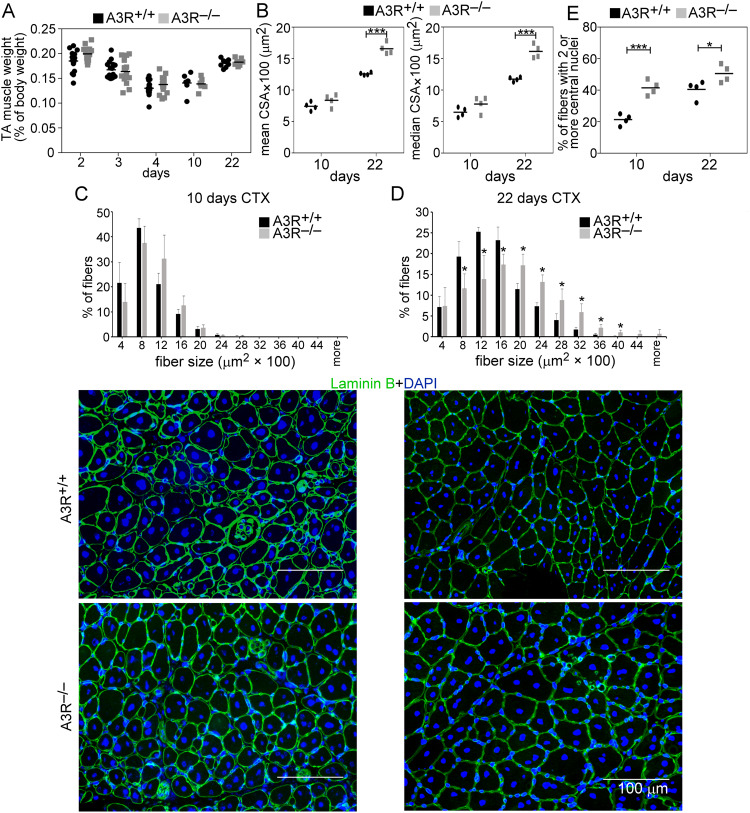
Increased muscle fiber size in the regenerating muscles of A3R^−/−^ mice. **A** Muscle/body weights, **B** mean and median myofiber cross-sectional areas (CSA), and **C**, **D** distribution of myofiber sizes in TA muscles of A3R^+/+^ and A3R^−/−^ mice at day 10 and 22 post-CTX–induced injury together with their representative immunofluorescence images of laminin (green) and DAPI (blue) nuclear staining. Scale bars, 100 µm. 500 or more myofibers were analyzed in each sample using ImageJ software. Data are expressed as mean or median ± SD. **E** Percentage of newly formed myofibers containing two or more central nuclei in TA muscles of A3R^+/+^ and A3R^−/−^ mice at day 10 and 22 post-CTX–induced injury. Data are expressed as mean or median and individual values in panels (**A**, **B**, and **E**) (*n* = at least 15 samples in panel (**A**) and *n* = 4 on panels (**B** and **E**)), while as mean ± SD (*n* = 4) in panels (**C** and **D**). Statistical significance was determined by two-tailed Student’s *t-*test. *p < 0.05, ***p < 0.001.

To characterize further the muscle regeneration in the absence of A3Rs, we compared the muscle weights and the myofiber CSAs of vehicle- and CTX-treated TA muscles from A3R^+/+^ and A3R^−/−^ mice. TA muscle weights were not different between control and regenerating muscles at day 10 or 22 post-injury in A3R^−/−^ mice as compared to the wild-type controls (Fig. 4A). The mean and median CSA of newly formed myofibers with central nuclei in A3R^−/−^ mice, however, were similar at day 10 (Fig. 4B, C), but significantly larger than in A3R^+/+^ mice at day 22 post-injury (Fig. 4B, D). The CSA frequency distribution showed similar fiber size distribution in control A3R^+/+^ and A3R^−/−^ mice, but the frequency of smaller fibers was significantly smaller, while that of bigger fibers was significantly higher in day 22 regenerating A3R^−/−^ muscles as compared to the wild-type ones (Fig. 4D).

The number of myofibers with two or more central nuclei is an indicator of myoblast fusion in the regenerating muscles. In accordance with the above data, the number of newly formed fibers with 2 or more central nuclei was enhanced in A3R^−/−^ mice as compared to wild-type mice both at day 10 and 22 post-injury (Fig. 4E). These data altogether indicated an acceleration in the skeletal muscle regeneration in the absence of A3Rs characterized by faster clearance of necrotic areas, by earlier removal of or less deposited collagen, by enhanced myoblast fusion, and by larger myofibers produced.

### Loss of A3Rs results in increased recruitment of CD45^+^ cells to the injury site

**Fig. 5:**
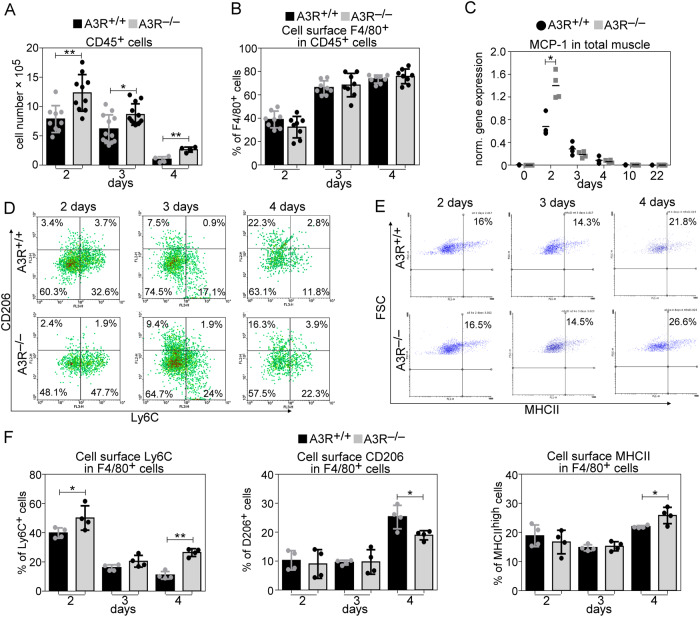
Increased leukocyte infiltration and altered macrophage polarization in regenerating A3R^−/−^ TA muscles. **A** Alterations in the number of CD45^+^ leukocytes per injured muscle and **B** percentage of anti-F4/80 antibody stained MΦs within the CD45^+^ leukocyte population in A3R^+/+^ and A3R^−/−^ TA muscles during the first 4 days of regeneration following CTX-induced injury (*n* = at least 4 samples). **C** MCP-1 mRNA expression levels in wild-type or A3R null TA muscles determined by RT-qPCR following CTX-induced injury (*n* = 4). **D** Representative scatter plots of CD206/Ly6C-stained and **E** MHCII^high^ muscle‐derived F4/80^+^ cells determined at the indicated days following CTX‐induced injury in the TA muscles of A3R^+/+^ and A3R^−/−^ mice. **F** Percentages of Ly6C^+^, CD206^+^, and MHCII^high^ cells within the muscle‐derived F4/80^+^ population determined at the indicated days following CTX‐induced injury in the TA muscles of A3R^+/+^ and A3R^−/−^ mice (*n* = 4). Data are expressed as mean and individual values, and statistical significance was determined by two-tailed Student’s *t-*test. *p < 0.05, **p < 0.01 (*n* = 4).

A3Rs are not expressed by the skeletal muscle [34] indicating that the observed changes are not related to the skeletal muscle itself. Since the migration of inflammatory cells to the injured area and tissue inflammation play a crucial role in the muscle regeneration process following injury [3, 4], our interest turned to the inflammatory cells. To assess the number of leukocytes and the percentage of MΦ population within the recruited cells in the early phase of muscle regeneration, we performed flow cytometric analysis of magnetically separated CD45^+^ cells from collagenase-digested muscles. In accordance with previous observations, we detected early infiltration of CD45^+^ cells at day 2 post-injury (Fig. 5A) and an increasing percentage of MΦs within this cell population at days 3 and 4 in wild-type mice (Fig. 5B). Loss of A3Rs did not affect the MΦ/CD45^+^ ratios (Fig. 5B), but the number of infiltrating CD45^+^ cells (Fig. 5A) and the level of monocyte chemoattractant protein-1 (MCP-1), a chemoattractant signal for both the neutrophil and MΦ recruitment [35, 36] (Fig. 5C), were significantly increased in the A3R^−/−^ regenerating muscle.

### Altered pro-inflammatory/reparative phenotypic switch in A3R^−/−^ macrophages during the muscle regeneration process

Since adenosine was reported to promote M1/M2 phenotypic change in MΦs [37], and proper phenotypic change is needed for proper tissue regeneration, we decided to determine the phenotypic switch of A3R^−/−^ MΦs during the skeletal muscle regeneration process. As demonstrated in Fig. 5D–F, we detected a delay in the disappearance of M1 specific [38–40] Ly6C^high^ and in the appearance of M2 specific CD206 phagocytic receptor [41] expressing F4/80^+^ MΦs in A3R^−/−^ muscles. In contrast, the appearance of MHCII^high^ expressing A3R^−/−^ F4/80^+^ MΦs [42] was enhanced.

**Fig. 6:**
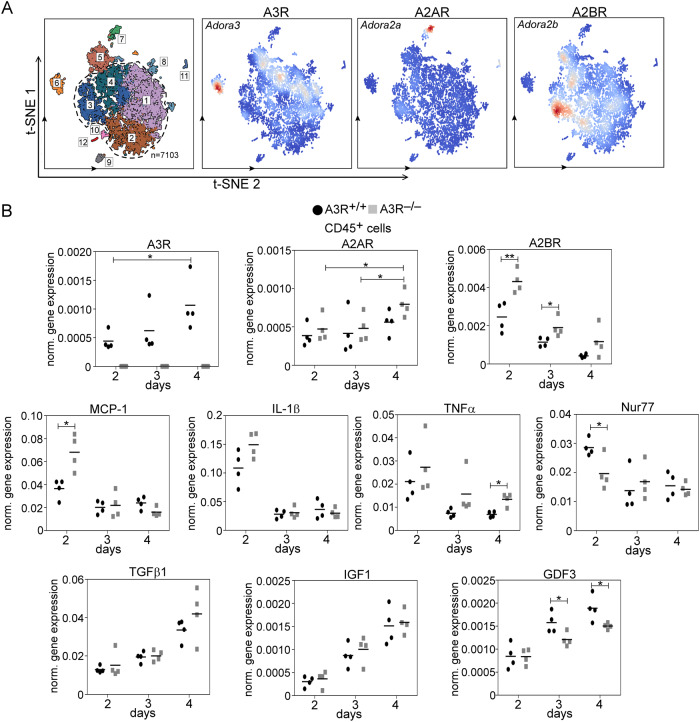
Loss of A3R impacts the gene expression of the muscle-derived CD45^+^ cells. **A** Single-cell analysis showing the expression of various adenosine receptors in CD45^+^ cells isolated from TA muscles of wild-type mice at day 4 post-CTX injury. 1. Resolution-related MΦs, 2. Growth factor-expressing MΦs, 3. Pro-inflammatory MΦs, 4. Antigen-presenting MΦs. 5. Neutrophils, 6. CD8a^+^ Clec9a^+^dendritic cells, 7. Fibroblasts, 8. Fibroblast-like cells, 9. Myeloid CD8a^-^ dendritic cells, 10. Endothelial cells, 11. T cells, NK-T cells, 12. Lymphoid CD8a^+^ dendritic cells. **B** mRNA expressions of various adenosine receptors and inflammatory markers in muscle-derived CD45^+^ cells isolated at various time points post-CTX injury detected by RT-qPCR. Data are expressed as mean and individual values and statistical significance was determined by two-tailed Student’s *t-*test and ANOVA. *p < 0.05, **p < 0.01 (*n* = 4).

Since our previous studies indicated that loss of such a protein, which is selectively expressed by a subgroup of reparative MΦs, can result in an imbalance in their phenotypic switch [22], we also looked at the expression of A3Rs in the various subpopulations of isolated CD45^+^ cells at day 4 following CTX injury (Fig. 6A). We found that its expression is low in pro-inflammatory MΦs, but is selectively upregulated within the resolution-related and antigen-presenting reparative MΦ subgroups. In addition, high expression was found in CD8^+^ Clec9a^+^ dendritic cells and neutrophils as well.

A3R expression was also found in both day 2 and day 3 muscle-specific CD45^+^ cells (Fig. 6B). As the percentage of macrophages increased within the CD45^+^cell population, the expression of A3Rs also increased. The expression of A2ARs also increased with time within the CD45^+^ cell population (Fig. 6B). Since A2AR was expressed at higher levels in pro-inflammatory macrophages, this increase might represent its elevated expression in the lymphoid CD8a^+^ dendritic cells (Fig. 6A). Loss of A3Rs did not affect significantly the expression of A2ARs, however, a significant compensatory increase was detected in the case of A2BRs (Fig. 6B). Unlike the expression of A3Rs, the expression of A2BRs was more dominant in the pro-inflammatory MΦs and neutrophils (Fig. 6A), and as both the percentage of neutrophils and of pro-inflammatory macrophages decreased with time during the regeneration process, the expression of A2BRs decreased concomitantly. Altogether these data indicate a signaling role of A2Rs in the pro-inflammatory, while that of A3Rs in neutrophils and in resolution-related and antigen-presenting MΦs.

In accordance with the in vivo muscle gene expression (Fig. 5C), at day 2 post-injury the expression of MCP-1 was significantly higher in the CD45^+^ cells as well (Fig. 6B) indicating that not only the increased number of transmigrating cells is responsible for its increased expression in the total muscle (Fig. 5C). Loss of A3Rs did not affect significantly the time-dependent changes in the expression of two pro-inflammatory cytokines (IL-1β and TNF-α), though their mRNA expression levels tend to be higher at day 2 post-injury. Since previous studies indicated that the Nur77 transcription factor could attenuate the pro-inflammatory responses in monocytes and MΦs [43], and adenosine was shown to induce Nur77 expression [44], we determined the mRNA expression of Nur77 as well. As shown in Fig. 6B, on day 2 post-injury we detected significantly lower Nur77 mRNA levels in A3R^−/−^ CD45^+^ cells as compared to their wild-type counterparts, but this difference disappeared by day 3. In contrast, the mRNA expressions of the M2-associated IGF-1 and TGF-β cytokines were not altered, but the expression of GDF3 significantly decreased from day 3 post-injury in A3R null cells as compared to the wild-type cells. Altogether these data demonstrated a delayed pro-inflammatory/reparative macrophage switch during skeletal muscle regeneration in A3R^−/−^ mice but more dominantly, an early enhanced pro-inflammatory response which was characterized by an attenuated Nur77 upregulation.

### Enhanced proliferation and earlier differentiation of SCs in the regenerating skeletal muscle of A3R null mice

To assess further the effect of the loss of A3Rs in skeletal muscle regeneration, the number of SCs, and the mRNA levels of myogenic genes, such as Pax7, and MyoD transcription factors involved in the regulation of SC proliferation and differentiation [45], and myogenin, a myoblast differentiation marker responsible for regulating myoblast fusion by transcribing its critical elements [46] were examined in the control and regenerating TA muscles.

**Fig. 7:**
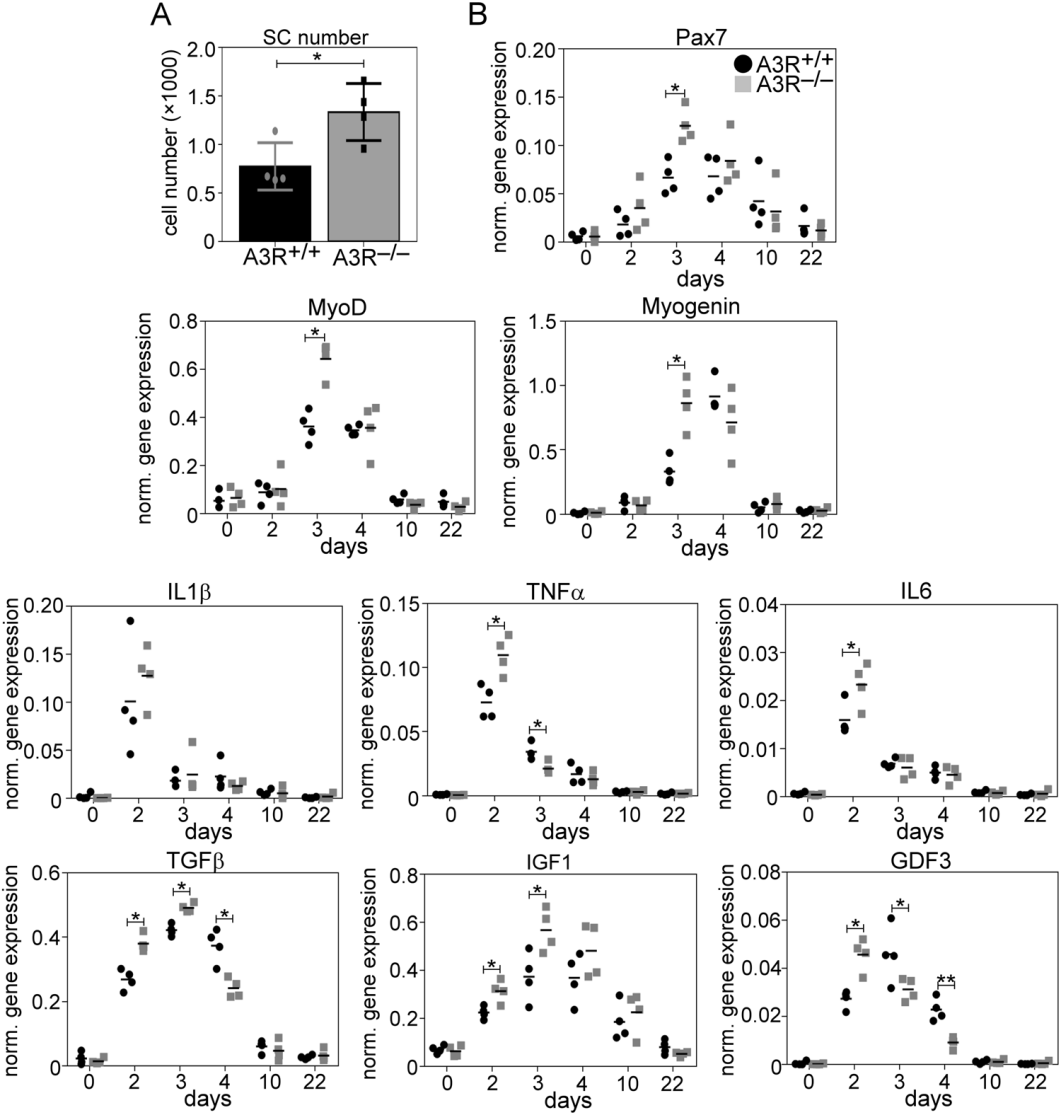
Enhanced proliferation and earlier differentiation of SCs in the regenerating skeletal muscle of A3R null mice. **A** Number of satellite cells isolated from regenerating TA muscles of A3R^+/+^ and A3R^−/−^ mice and expression of Pax7 in control and regenerating TA muscles detected by RT-qPCR. **B** Expression of myogenic and inflammatory markers in control and regenerating TA muscles isolated from A3R^+/+^ and A3R^−/−^ mice at various time points post-injury detected by RT-qPCR. Data are expressed as mean and individual values, and statistical significance was determined by two-tailed Student’s *t-*test. *p < 0.05, **p < 0.01 (*n* = 4).

The transcription factor Pax7 is considered to be a canonical quiescent SC marker, and its expression is maintained during the progression to SC activation and proliferation but is downregulated at the onset of myogenic differentiation [47]. Thus, its mRNA expression correlates with the number of SCs. As shown in Fig. 7A, at day 4 post-injury, we detected a significantly increased number of SCs in the A3R^−/−^ TA muscle, as compared to their wild-type littermates. Accordingly, a significantly increased Pax7 mRNA expression was detected in the A3R^−/−^ muscles on day 3 following injury (Fig. 7B). The expression of MyoD increased proportionally with that of Pax7 by day 3 in A3R^−/−^ muscles indicating that its enhanced expression is related to the enhanced SC numbers rather than to an increased expression within the SCs. The mRNA expression of myogenin, however, reached its peak at day 4 post-injury in the wild-type muscles, as we have reported previously [48], but already at day 3 in the A3R^−/−^ muscle. These data indicated an enhanced proliferation and then an earlier start of differentiation of SCs into myoblasts with a concomitant earlier drop in the Pax7 gene mRNA expression in the A3R^−/−^ muscle.

Next, we determined the mRNA expressions of several cytokines and growth factors known to influence the proliferation and differentiation of SCs, since their expressions in the total muscle better reflect their in vivo production to which the myoblasts are exposed. In accordance with the increased number of transmigrating inflammatory cells, at day 2 post-injury a significantly increased mRNA expression of pro-inflammatory cytokines, such as IL-1β, TNF-α, or-IL-6, all known to be produced by neutrophils as well [49], was detected in the A3R^−/−^ muscles. All these signaling molecules were shown to promote SC proliferation [11, 13]. By day 3 post-injury the mRNA expression levels of these cytokines started to decrease. Concomitantly, that of GDF3, an early inhibitor of myoblast differentiation [18], also decreased significantly allowing the early initiation of differentiation. At day 3 post-injury, the mRNA expressions of factors that promote differentiation (such as TGF-β or IGF-1) reached their peak, and especially that of IGF-1 known to regulate myogenin expression [47] was expressed at a significantly higher level in the A3R^−/−^ muscles than in the wild-type ones. All these alterations in the cytokine expressions might contribute to the faster proliferation, to the earlier differentiation of SCs, and to the enhanced myoblast fusion in the A3R^−/−^ muscles.

### The accelerated skeletal muscle regeneration of A3R^−/−^ mice is not related to the loss of A3Rs in the bone marrow-derived cells

**Fig. 8:**
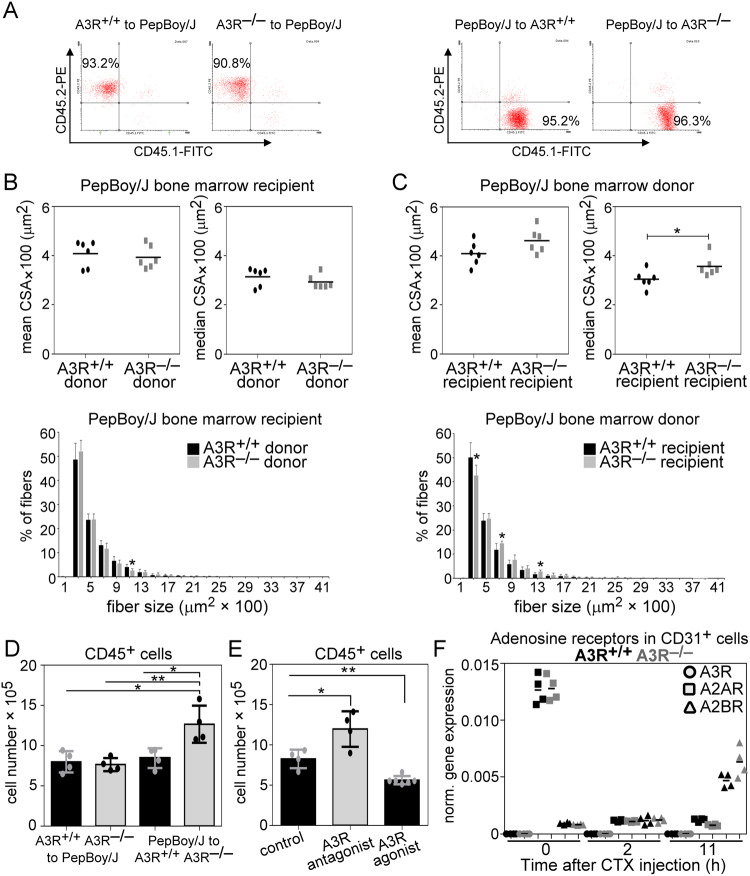
Tissue-resident cells mediate the increase in muscle fiber size in the A3R^−/−^ TA muscle. **A** Representative scatter plots of CD45.1 and CD45.2 stained blood-derived leukocytes from bone marrow-transplanted mice. **B** Mean and median CSA values and fiber size repartition of 22-day regenerated TA muscles isolated from wild-type mice transplanted with wild-type or A3R^−/−^ bone marrow. **C** Mean and median CSA values and fiber size repartition of 22-day regenerated TA muscles isolated from wild-type or A3R^−/−^ mice transplanted with wild-type bone marrow. **D** Number of muscle-infiltrating CD45^+^ cells isolated from the regenerating TA muscles of bone marrow-transplanted mice on day 2 post-CTX-injury. **E** Number of muscle-infiltrating CD45^+^ cells on day 2 post-CTX-injury in wild-type TA muscles co-injected either with MRS1191, an A3R antagonist (2 mg/kg), or IB- MECA, an A3R agonist (100 μg/kg). **F** Expression of adenosine receptors in muscle-derived CD31^+^ cells at various times following CTX-induced injury determined by RT-qPCR. Mean and individual values are shown, and statistical significance was determined by two-tailed Student’s *t-*test and ANOVA. *p < 0.05, **p < 0.01 (*n* = 6 in panels (**B** and **C**), *n* = 4 in panels (**D**, **E** and **F**)).

If the loss of A3Rs, which influences skeletal muscle regeneration, affects primarily the transmigrating cells, then the loss of A3Rs in the bone marrow-derived cells should also affect the skeletal muscle regeneration. To test this possibility, wild-type or A3R^−/−^ mice were terminally irradiated, and their bone marrow was replaced by bone marrow originating from either wild-type or A3R^−/−^ mice in each combination. To confirm successful ablation and reconstitution, we involved wild-type PePBoy/J (a CD57BL/6 variant strain) mice in these experiments, which express the CD45.1 allelic variant of CD45, while C57BL/6 mice are CD45.2 positive. Hematologic and flow cytometric analysis of these mice following bone marrow transplantation demonstrated a leukocyte repopulation of over 90% (Fig. 8A).

To our surprise, while loss of A3Rs from bone marrow-derived cells did not affect the skeletal muscle regeneration (Fig. 8B), a significantly increased fiber size was detected at day 22 post-injury if A3Rs were missing from the tissue-resident cells (Fig. 8C). To test whether loss of A3Rs affects transmigration of inflammatory cells if they are lost separately in the bone marrow-derived or in the tissue-resident cells, the transmigration of CD45^+^ leukocytes was also determined in the above setup two days following CTX-induced injury (Fig. 8D). Irradiation and bone marrow transplantation did not affect the number of transmigrating cells if wild-type animals were transplanted with wild-type cells, in accordance with a previous report [50]. Neither did the number alter if wild-type animals were transplanted with A3R^−/−^ cells. When, however, the recipient was A3R deficient, an increased number of transmigrating cells was detected. These observations indicated that tissue-resident cells affect primarily the magnitude of inflammatory cell transmigration, which then drives accelerated skeletal muscle regeneration.

Since the loss of A3Rs leads to increased A2BR expression (Fig. 5A), we decided to test whether the loss of A3Rs or the compensatory increase in A2BR expression leads to the increased transmigration of inflammatory cells. As seen in Fig. 8E, similar to the loss of A3Rs, an A3R antagonist promoted, while an A3R agonist inhibited the transmigration of CD45^+^cells in wild-type animals detected at day 2 following CTX-induced injury, underlying the determining role of A3Rs.

Since among the tissue-resident cells, capillary endothelial cells are known to affect the transmigration of the inflammatory cells [51], we determined their adenosine receptor mRNA expressions. As seen in Fig. 8F, while we could readily detect the expression of A2A and A2BRs, the expression of A2ARs decreased, while that of A2BRs increased with time following CTX-induced injury, we found no evidence for A3R expression. Thus, we exclude that loss of A3Rs from the capillary endothelial cells would be responsible for the enhanced transmigration of inflammatory cells.

## Discussion

Adenosine has been known for a long time as an endogenously produced signaling molecule to negatively regulate inflammation [23]. Since regenerative inflammation plays a determinant role in regulating tissue regeneration following injury, in the present study the involvement of A3Rs was investigated in the regeneration of the TA muscle following CTX-induced injury. Our data demonstrate that though skeletal muscle cells do not express A3Rs [34], loss of A3Rs leads to their faster regeneration and results in a regenerated skeletal muscle containing larger myofibers.

Loss of A3Rs induced an enhanced early pro-inflammatory milieu formation in the TA muscle following CTX-induced injury, characterized by increased transmigration of inflammatory cells, by enhanced pro-inflammatory cytokine, and earlier IGF-1 production on the muscle level, and by a delayed M1/M2 conversion of MΦs.

The transmigrating cells appearing in increased numbers in the injured muscle of A3R^−/−^ mice were able to clear the cell debris more efficiently than their wild-type counterparts. Since it is believed that necrotic myofibers may act as either atrophic factors to repress myoblast growth or physical barriers to prevent myoblast fusion, enhanced engulfment of dead cells by phagocytes enabled a more efficient skeletal muscle regeneration by giving the possibility for an earlier start of the repair processes [52]. All these events together lead to an increase in the SC proliferation, to an earlier and higher expression of myogenin responsible for inducing the machinery for myoblast fusion [46], and consequently to an enhanced myoblast fusion and generation of larger myofibers. Our data are in harmony with previous reports, which indicated that inflammation influences tissue regeneration following injury [53]. However, enhanced inflammation might lead to further injury, thus, proper balance in the magnitude of inflammation and in the tissue regeneration response must be maintained [3, 4]. Our data demonstrate that in the context of skeletal muscle regeneration A3Rs are anti-inflammatory, and interestingly, the higher inflammatory response observed in the absence of A3Rs remained in the range where it had a regeneration-promoting effect.

Since previous studies indicated that triggering A3Rs has an anti-inflammatory effect on both MΦs [25] and neutrophils [54], we expected that the loss of A3Rs affects primarily the bone marrow-derived cells. To our surprise, it was not the case. Loss of A3Rs affected primarily the tissue-resident cells, and these cells were responsible for recruiting increased numbers of inflammatory cells following injury. Capillary endothelial cells, tissue-resident macrophages and mast cells can all contribute to the increased transmigration of inflammatory cells. Since, however, we did not detect A3Rs in the capillary endothelial cells of the TA muscle, we hypothesize that the inflammatory response to tissue injury of tissue-resident macrophages [25] and/or mast cells [55] is the one that is negatively regulated by the A3Rs. In addition to creating an enhanced inflammatory milieu, these cells might have contributed to the enhanced SC proliferation also by interacting with the quiescent SCs promoting their activation [9, 10]. Unfortunately, we had no tool to prove this later possibility. Altogether our data indicate that the A3Rs are negative regulators of injury-related regenerative inflammation and consequently also that of the muscle fiber growth in the TA muscle. Thus, inhibiting A3Rs might have a therapeutic value during skeletal muscle regeneration following injury.

### Supplementary information

Supplementary Figure 1

Supplementary Figure 1 legend

Supplementary table 1

Supplementary table 2

## Data Availability

The scRNA-seq data used in this study are publicly available in the GEO under accession number GSE161467 [19]. The rest of the data generated or analyzed during this study are available from the corresponding author upon reasonable request.
