# Supplementary figures and images for: Loss of adenosine A3 receptors accelerates skeletal muscle regeneration in mice following cardiotoxin-induced injury
Source: Cell Death Dis. 2023 Oct 28;14(10):706. doi: 10.1038/s41419-023-06228-7 (PMC10613231; doi:10.1038/s41419-023-06228-7)

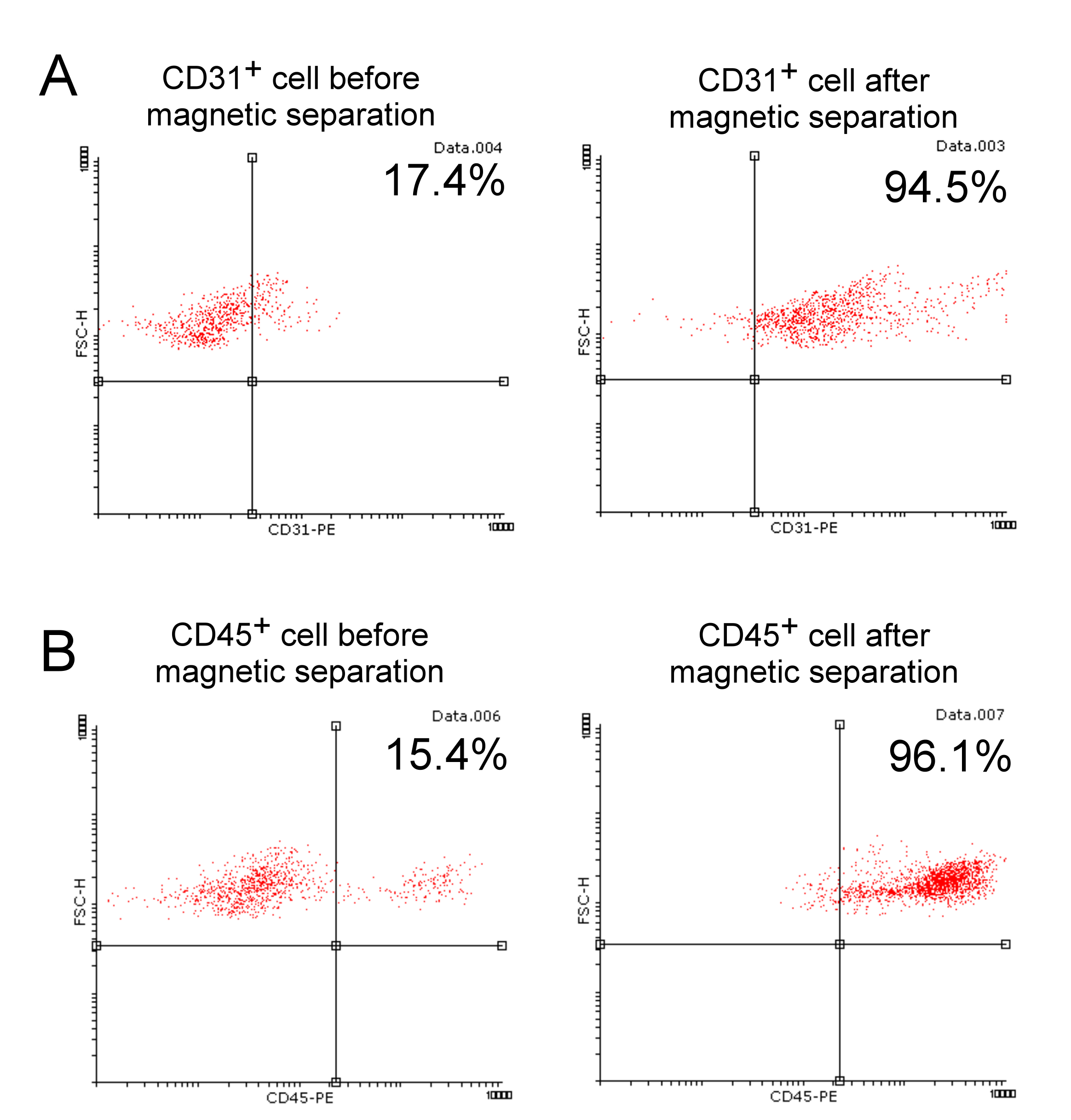

Supplement: Supplementary file 1 — Supplementary Figure 1 [file 41419_2023_6228_MOESM1_ESM.tif]
