# Supplementary material for: Loss of adenosine A3 receptors accelerates skeletal muscle regeneration in mice following cardiotoxin-induced injury
Source: Cell Death Dis. 2023 Oct 28;14(10):706. doi: 10.1038/s41419-023-06228-7 (PMC10613231; doi:10.1038/s41419-023-06228-7)
Supplement: Supplementary file 2 — Supplementary Figure 1 legend [file 41419_2023_6228_MOESM2_ESM.docx]

**Supplementary figure legends**

**Fig. S1. Purity of CD45^+^ or CD31^+^cells separated by magnetic beads following cardiotoxin-induced injury.** Muscle injury was induced by injecting 50 μl of 12 μM cardiotoxin (CTX) into the tibialis anterior (TA) muscle of wild type mice. **A** CD31^+^ cells were isolated after 11 h, while **B** CD45^+^ cells after 3 days following CTX-induced injury. The purity of isolated cells was determined by FACS analysis following staining them them by phycoerythrin-conjugated antibodies against mouse CD31 and CD45.2, respectively.
