# Supplementary material for: Loss of adenosine A3 receptors accelerates skeletal muscle regeneration in mice following cardiotoxin-induced injury
Source: Cell Death Dis. 2023 Oct 28;14(10):706. doi: 10.1038/s41419-023-06228-7 (PMC10613231; doi:10.1038/s41419-023-06228-7)
Supplement: Supplementary file 3 — Supplementary table 1 [file 41419_2023_6228_MOESM3_ESM.pdf]

Supplementary table 1: A3R$^{-/-}$ mice are characterized by decreased grip force and voluntary running speed.

Grip force	A3R$^{+/+}$ (n = 5)	A3R$^{-/-}$ (n = 5)
Body weight (g)	27.96 ± 0.28	26.9 ± 0.74
Maximal force (mN)	161.94 ± 7.13	122.79 ± 3.44**
Force normalized to body weight (nM/g)	5.8 ± 0.27	4.57 ± 0.07**
Voluntary running	A3R$^{+/+}$ (n = 4)	A3R$^{-/-}$ (n = 5)
Distance (m/day)	7805 ± 262.6	8002.8 ± 201.7
Average speed (m/min)	13.2 ± 0.5	11.6 ± 0.2*
Max speed (m/min)	24.9 ± 0.8	19.3 ± 0.4***
Forced running	A3R$^{+/+}$ (n = 5)	A3R$^{-/-}$ (n = 5)
Time (min)	15.55 ± 0.48	16.8 ± 1.0
Distance (m/day)	347.6 ± 13.6	386.5 ± 31.3

Mean values ± SD are shown, and statistical significance was determined by two-tailed Student's *t* test. *p < 0.05, ** p < 0,01, and *** p < 0.001.
