# Supplementary material for: Loss of adenosine A3 receptors accelerates skeletal muscle regeneration in mice following cardiotoxin-induced injury
Source: Cell Death Dis. 2023 Oct 28;14(10):706. doi: 10.1038/s41419-023-06228-7 (PMC10613231; doi:10.1038/s41419-023-06228-7)
Supplement: Supplementary file 4 — Supplementary table 2 [file 41419_2023_6228_MOESM4_ESM.pdf]

Supplementary table 2: Normal EDL and increased SOL muscle force in the absence of A3R

EDL muscle	TWITCH		TETANUS	
	A3R$^{+/+}$ (n = 10)	A3R$^{-/-}$ (n = 10)	A3R$^{+/+}$ (n = 10)	A3R$^{-/-}$ (n = 10)
EDL Force (mN/mm^2)	1.6 ± 0.19	1.59 ± 0.12	7.24 ± 0.76	7.35 ± 0.47
SOL Force (mN/mm^2)	1.87 ± 0.16	2.46 ± 0.21*	9.21 ± 0.64	12.22 ± 1.14*

Mean values ± SD are shown, and statistical significance was determined by two-tailed Student's *t* test. *$p < 0.05$.
